# Lyophilized Extracellular Vesicles from Adipose-Derived Stem Cells Increase Muscle Reperfusion but Degrade Muscle Structural Proteins in a Mouse Model of Hindlimb Ischemia-Reperfusion Injury

**DOI:** 10.3390/cells12040557

**Published:** 2023-02-09

**Authors:** Bharati Mendhe, Mohammad B. Khan, Damon Dunwody, Khairat Bahgat Youssef El Baradie, Kathryn Smith, Wenbo Zhi, Ashok Sharma, Tae Jin Lee, Mark W. Hamrick

**Affiliations:** 1 Medical College of Georgia, Augusta University, Augusta, GA 30912, USA; 2 Faculty of Science, Tanta University, Tanta 31111, Egypt; 3 College of Medicine, University of Arkansas for Medical Sciences, Little Rock, AR 72205, USA

**Keywords:** annexin a1, Hsp90, mitochondrial permeability, exosomes, dystrophin

## Abstract

Ischemia-reperfusion (I/R) injury is a complication impacting multiple organs and tissues in clinical conditions ranging from peripheral arterial disease to musculoskeletal trauma and myocardial infarction. Stem cell-derived extracellular vesicles (EVs) may represent one therapeutic resource for preventing the tissue damage associated with I/R injury. Here we tested the hypothesis that lyophilized extracellular vesicles derived from adipose stem cells could serve as an “off-the-shelf” treatment modality for I/R injury in a mouse hindlimb ischemia model. Ischemia was induced for 90 min using a rubber band tourniquet and extracellular vesicles (0, 50, or 100 µg) administered via tail vein injection immediately prior to reperfusion. Perfusion was measured prior to, during, and after ischemia using laser Doppler imaging. Serum and tissue were collected 24 h after reperfusion. Mass spectrometry (MS)-based proteomics was used to characterize the EV cargo and proteins from the ischemic and non-ischemic hindlimb. Inflammatory cytokines were measured in muscle and serum using a multiplex array. Results indicate that EVs significantly increase reperfusion and significantly increase expression of the anti-inflammatory factor annexin a1 in skeletal muscle; however, the increased reperfusion was also associated with a marked decrease in muscle structural proteins such as dystrophin, plectin, and obscurin. Circulating inflammatory cytokines TNF-alpha and IL-6 were increased with EV treatment, and serum TNF-alpha showed a significant, positive correlation with reperfusion level. These findings suggest that, while EVs may enhance reperfusion, the increased reperfusion can negatively impact muscle tissue and possibly remote organs. Alternative approaches, such as targeting mitochondrial permeability, may be more effective at mitigating I/R injury.

## 1. Introduction

Ischemia-reperfusion (I/R) injury is a complication impacting multiple organs and tissues in clinical conditions ranging from peripheral arterial disease to knee arthroplasty, musculoskeletal trauma, and myocardial infarction. The general sequelae of cellular-level events in I/R are becoming better understood. The period of anoxia is followed by reperfusion, which generates reactive oxygen species (ROS) and accumulation of calcium (Ca ^2+^) in mitochondria. ROS accumulation and calcium overload lead to mitochondrial swelling and sustained opening of the mitochondrial permeability transition pore (mPTP) [1,2]. These changes in mitochondria lead to infolding of the inner mitochondrial membrane, outer mitochondrial membrane rupture, cell damage, and cell death. Cell damage and ROS release from the mPTP stimulate the expression and secretion of inflammatory cytokines, which circulate to induce remote tissue damage [3].

Several therapeutic approaches for preventing I/R injury have been studied. These range from remote ischemic conditioning to small molecule therapies that target the mPTP [4,5]. In recent years, cell therapies have been explored utilizing stem cell-derived extracellular vesicles. Several of these studies have shown that EVs can increase angiogenesis following ischemia and, in so doing, can also increase reperfusion [6,7,8,9]. Indeed, we have recently found in an in vitro model that EVs derived from mesenchymal stem cells can promote muscle cell survival and reduce toxicity [10]. The practical application of EVs as a therapeutic will require storage approaches that can retain the integrity of EV cargo, and long-term storage is known to decrease the bioactivity of EVs [11,12]. We used a cryopreservation approach employing trehalose and polyvinylpyrrolidone (PVP) to improve the stability and bioactivity of stem cell-derived EVs [10]. Here we test the hypothesis that lyophilized EVs can attenuate muscle damage following I/R injury in a mouse hindlimb I/R model.

## 2. Materials and Methods

### 2.1. Exosome Isolation and Preparation

Mouse Adipose-Derived mesenchymal stem cells (mADSCs) (CD29, CD44, Sca-1 positive and CD117, CD31 negative) purchased from Cyagen, catalog number MUBMD-01001, lot number Lot: 190901G01 (US Inc. 2255 Martin Ave., Suite E Santa Clara, CA 95050, USA). The cells were cultured in Dulbecco’s Modified Eagle Medium (DMEM/High Glucose; Hyclone, Logan, UT, USA), supplemented with 10% fetal bovine serum and 1% penicillin/streptomycin. The mADSCs were cultured at 37 °C under 5% CO_2_ and used at passages 4–6 in all experiments. mADSCs EVs were isolated as per our published method [10]. We used between 2–5 cell passages to isolate the EVs. In brief, mADSCs were seeded in 150 mm dishes within DMEM/High Glucose, supplemented with 10% FBS and 1% penicillin/streptomycin. After cells reached ∼80% subconfluence, cells were washed with 1X PBS and cultured for 24 h with 25 mL of DMEM media with 5% exosome-depleted FBS. Conditioned medium was collected after 24 h, and 25 mL of fresh medium was added to the culture dish for another 24 h. The conditioned medium was collected from each dish again and mixed with media collected previously to obtain 50 mL of the conditioned medium. 

Exosomes were isolated from the conditioned medium using tangential fluid filtration (TFF) system (EMD Millipore Corporation, Billerica, MA, USA) [10]. The conditioned medium was collected and centrifuged at 10,000× *g* for 30 min to separate cell debris. The supernatant was collected and filtered through a 0.2 µm Nalgene PES membrane filter (Thermo Fisher Scientific, Waltham, MA, USA). After filtration, the conditioned medium was then subjected to ultrafiltration in a Lab-scale Tangential Flow Filtration System using a 300 kDa Pellicon XL Cassette. A feed flow rate of 30–50 mL/min, transmembrane pressure < 3.5 psi, and crossflow rate > 10:1 were maintained throughout the filtration process. After the conditioned medium was concentrated 10-fold, the diafiltration step was started by changing the media with 1X PBS. The concentrated EVs were lyophilized in 100 mM trehalose + 5% PVP40 [10]. Particle size averaged 169 nm, and the concentration did not differ significantly from samples isolated by ultracentrifugation that were not lyophilized [10]. The lyophilized EV samples were stored at room temperature for one month before experimental testing. They were rehydrated with ddH_2_O by adding the original volume and then vortexed before use.

### 2.2. Exosome Protein Extraction and Digestion for LC-MS Analysis

LC-MS analysis was used to characterize the EV cargo. 100 µL freshly made 50 mM ammonium bicarbonate buffer with 0.1% (*w/v*) RapiGest SF Surfactant (Waters), and 10 mM dithiothreitol was added into sample tubes to resuspend the exosome sample and reduce the disulfide bonds at 60 °C for 30 min. The samples were then alkylated by iodoacetamide in the dark for 30 min, followed by digestion for 16 h using trypsin (Thermo Scientific #90057) at 37 °C. Trifluoroacetic acid was added to the sample tube to a final concentration of 0.1% (*v/v*) to stop the digestion. The samples were then incubated at 37 °C for 40 min to cleave the detergent. The samples were centrifuged at 15,000× *g* for 5 min, and the supernatants were transferred into sample vials for LC-MS analysis. Results of the LC-MS analysis are provided in Table S1.

### 2.3. Ischemia-Reperfusion Experiments

**Figure 1:**
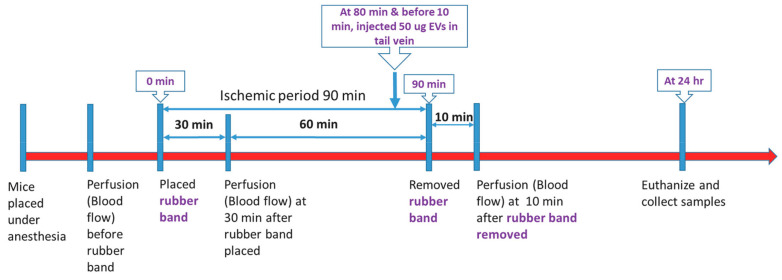
Experimental design. Hindlimb ischemia was induced for 90 min using an orthodontic rubber band. Blood flow was measured prior to band placement, after band placement, and after band removal using Laser Doppler imaging. EVs were delivered via a tail vein 10 min prior to reperfusion, and tissue was collected 24 h after reperfusion.

We utilized a rubber band tourniquet model of single-hindlimb ischemia as described previously [5,13]. All animal procedures employed during this study were reviewed and approved by the Augusta University Institutional Animal Care and Use Committee and are consistent with AAALAC guidelines. Measures were taken to minimize pain or discomfort in accordance with the Guide for the Care and Use of Laboratory Animals, 11th edition, National Research Council, 2011. Significant efforts were also made to minimize the total number of animals used while maintaining statistically valid group numbers. In brief, 40 CD-1 mice (28–35 g) (Envigo RMS, Inc., Indianapolis, IN, USA; 20 males and 20 females) were randomly allocated into four groups of ten mice each (5 males, 5 females): Group 1 (Control group, low dose EV vehicle), Group 2 (Treatment, low dose EV), Group 3 (Control group, high dose EV vehicle), Group 4 (Treatment, high dose EV). The vehicle treatments for the low and high-dose groups utilized the same solutions. We included a separate vehicle group for each treatment group to control for any possible variation between experiments. All mice in each of the four groups were included in the following ischemia-reperfusion experiments. Isoflurane was delivered by face mask at a concentration of 2% for induction and then 1% for maintenance. Continuous oxygen was provided at 2 L/min. Mice were kept on a heating pad to maintain the body temperature at 37 °C. Fur was removed from the left hindlimb with a hair removal cream and the skin was cleaned with 70% ethanol while mice were under anesthesia. Ischemia was induced on the proximal thigh with a 4.5 oz orthodontic rubber band (American Orthodontics, Sheboygan, WI, USA). Mice received either vehicle (PBS), low dose EVs (50 µg), or high dose EVs (100 µg) injected via tail vein in a total volume of 100 µL 10 min before tourniquet removal (Figure 1). We injected the EVs prior to tourniquet removal to determine whether or not they could attenuate the very rapid changes in muscle, such as increased ROS and Ca^+^ levels, that occur immediately after reperfusion. A Laser Doppler imager (Moor Instruments, Wilmington, DE, USA) was used to assess limb perfusion prior to ischemia, 30 min after placement of the tourniquet, and 10 min after removing the tourniquet (Figure 1) as described previously [5]. Briefly, the Laser Doppler source was mounted on a movable rack 10 cm above the mouse hindlimb. The laser beam (780 nm) was used to detect blood movement and then generate a computerized, color-coded image. Tissue perfusion was calculated from the relative flux (in U/cm^2^) in the areas corresponding to the plantar aspect of the hindlimb using image analysis software (Laser Doppler perfusion measure, V3.08, Moor Instruments). A flux unit is a unit of blood flow along a blood vessel, which is proportional to the volume of blood per unit of time that flows past a given point. Change in flux units, therefore, represents a change in relative perfusion.

### 2.4. Sample Collection and Tissue Analysis

Mice were euthanized by isoflurane overdose and cardiac exsanguination 24 h after reperfusion. Blood was collected and placed on ice for 2–3 h, centrifuged for 30 min at 1000 rpm, and serum was collected for cytokine array. Skeletal muscle tissue samples collected after euthanasia included tibialis anterior (TA) and gastrocnemius. TA muscles were fixed in 10% neutral buffered formalin and prepared for histology. Gastrocnemius muscles were flash-frozen in liquid nitrogen and stored at − 80 °C for cytokine array and proteomics. TA muscles were paraffin-embedded and cross-sections cut at the 2–3 μm thickness and stained with hematoxylin and eosin. Stained slides were examined for tissue damage, edema, and cellular infiltrate. While we did not use an immunohistochemical marker for neutrophils, the cellular infiltrate in mouse hindlimb muscle after ischemia and reperfusion is most commonly identified as composed primarily of neutrophils [14]. 

### 2.5. Analysis of Inflammatory Cytokines

Cytokine Bead Array Mouse Inflammation 13-Plex Panel (Bio-Legend, San Diego, CA, USA) was performed on mouse serum and half of each gastrocnemius muscle lysates in the Georgia Cancer Center Immune Monitoring Core accordingly to the manufacturer’s instructions.

### 2.6. Proteomic Analysis of Skeletal Muscle

The remaining portion of each gastrocnemius was homogenized in 500 µL ice-cold lysis buffer (8 M urea in 50 mM Tris-HCl (pH 8) with 1/100 protease inhibitor cocktail (Thermo Scientific #1861279)) and 0.2 mL mixed stainless steel beads at speed 10 for 3 min at 4 °C using a bullet blender (Next Advance, Inc.). The lysates were then centrifuged at 16,000× *g* at 4 °C for 15 min, and the supernatant was transferred to a new tube. The protein concentration of the lysate supernatant was measured using a Pierce™ Coomassie (Bradford) protein assay kit (Thermo Scientific #23200). A total of 50 µg of extracted protein was reduced with dithiothreitol, alkylated using iodoacetamide, and diluted 10-fold with 50 mM ammonium bicarbonate buffer before being digested overnight using trypsin at a 1:20 (*w/w*) ratio (Thermo Scientific #90057). Digested peptides were cleaned using a C18 spin column (Harvard Apparatus #744101) and then lyophilized.

The digested samples were separated via an Ultimate 3000 nano-UPLC system (Thermo Scientific) and analyzed with an Orbitrap Fusion Tribrid mass spectrometer (Thermo Scientific). The peptide mixture was washed on a Pepmap100 C18 trap (5 μm, 0.3 × 5 mm) for 10 min at a rate of 20 μL/min using 2% acetonitrile in water with 0.1% formic acid. The peptides were then separated on a Pepman100 RSLC C18 column (2.0 μm, 75 μm  × 150 mm) using a gradient of 2 to 40% acetonitrile with 0.1% formic acid over 120 min (flow rate: 300 nL/min; column temperature: 40 °C). Eluted peptides were analyzed via data-dependent acquisition in positive mode using the following settings: Orbitrap MS analyzer for precursor scan at 120,000 FWHM from 300 to 1500 m/z; Ion-trap MS analyzer for MS/MS scans in top speed mode (2-s cycle time) with dynamic exclusion settings (repeat count: 1; repeat duration: 15 s; exclusion duration: 30 s). The fragmentation method was higher-energy collision dissociation (HCD) with a normalized collision energy level of 30%.

Raw MS peptide data were analyzed using Proteome Discoverer (v1.4; Thermo Scientific) and then searched against the UniProt mouse protein database using TurboSequest. The following search parameters were utilized: 0.6 Da production tolerance, 10 ppm precursor size, 57.021 Da static carbidomethylation for cysteine 15.995 Da dynamic oxidation for methionine. Proteins were grouped if they had comparable peptide characteristics. Peptide spectrum matching (PSM) was validated using the Percolator PSM validator algorithm. PSM counts were normalized by comparing total PSM counts between samples.

### 2.7. Statistical Analysis

Between-group comparisons were performed on histological, perfusion, and cytokine data (IBM SPSS software v.28) using two-factor ANOVA with treatment and sex as the two factors. Post-hoc Fisher’s LSD tests were performed for pairwise comparisons, and regression analysis was performed between perfusion and cytokine data. For proteomic analysis, statistical analyses were performed using the R project for Statistical Computing (version 3.6.3). PSM values were log-transformed to achieve normal distribution. LIMMA package in R was utilized to compare treatment effects on the protein levels, and *p*-values were adjusted using a false discovery rate (FDR). The threshold for significance was set at an adjusted *p*-value  <  0.05.

## 3. Results

### 3.1. Proteomic Characterization of EV Cargo

Proteomic analysis of the lyophilized EVs indicates that they share several proteins in common with fresh EVs from ADSCs (Table 1). Approximately a third of these shared proteins are also commonly found in exosomes from a wide range of sources, including urine, plasma, liver, brain, and bone marrow (Table 1). These common EV-related proteins include moesin, gelsolin, fibronectin-1, HSP-90 alpha, and others (Table 1). 

### 3.2. Effects of EV Treatment on Muscle Histology, Reperfusion, and Inflammatory Cytokines

**Figure 2:**
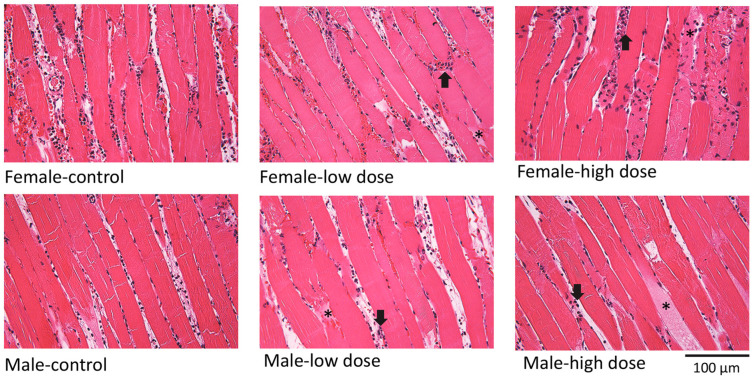
Effects of EV treatment on muscle morphology after I/R injury. Sections stained with hematoxylin and eosin show muscle fiber damage (asterisks) and cellular infiltration (arrows) with both low and high-dose treatment in male and female mice.

Tissue histology (Figure 2) shows that EV treatment was associated with muscle damage and cellular infiltration in both male and female mice at low and high doses.

Our experiments utilizing low-dose (50 µg) EV treatment did not result in any significant changes in hindlimb reperfusion (Table 2). This absence of any change in reperfusion was also associated with no significant treatment effects for muscle or circulating cytokine levels (Table 2).

**Figure 3:**
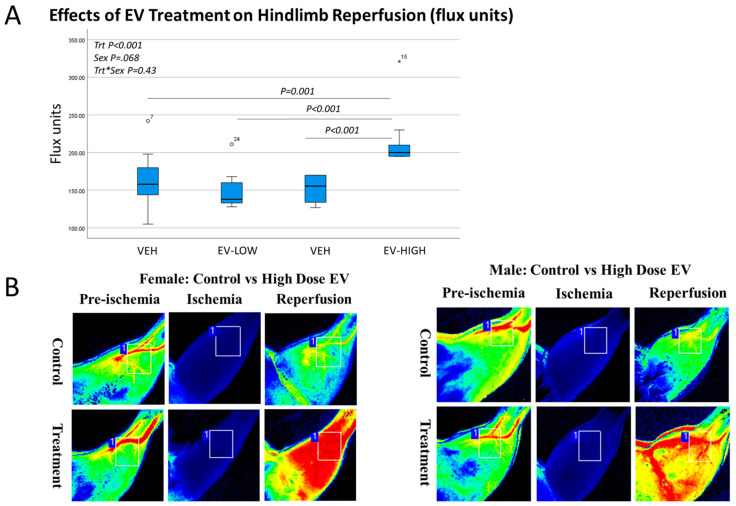
Effects of EV treatment on hindlimb reperfusion. (**A**). Box-and-whisker plots showing a significant increase in reperfusion with the high-dose EV treatment. Two-factor ANOVA was performed with treatment and sex as the two factors (*n* = 5 male, 5 female per group). (**B**). Laser Doppler images of hindlimbs from male and female mice receiving vehicle (control) or high dose (100 µg) EVs showing increased perfusion in the treated mice. °,* represent outliers in (**A**).

**Figure 4:**
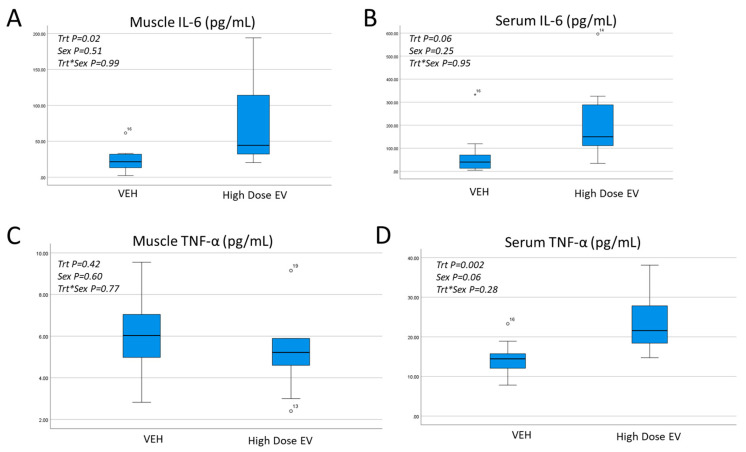
Effects of EV treatment on local and circulating inflammatory cytokines. Muscle-derived IL-6 (**A**) is increased significantly with EV treatment, which is associated with a trend toward higher serum IL-6 (**B**). Muscle-derived TNF-alpha (**C**) is not significantly increased with EV treatment, but serum TNF-alpha is significantly higher in EV-treated mice (**D**). Two-factor ANOVA was performed with treatment and sex as the two factors (*n* = 5 male, 5 female per group). °,* represent outliers.

Our experiments utilizing high-dose (100 µg) EV treatment did, however, result in a significant (+25%) increase in hindlimb reperfusion (Figure 3A). This difference was significant for males and females (Figure 3B). Cytokine array data show that the high-dose EV treatment was associated with increased muscle-derived IL-6 and a trend toward increased serum IL-6 (Figure 4A,B). Muscle-derived TNF-alpha was similar between groups, but the high-dose EV treatment significantly increased circulating TNF-alpha (Figure 4C,D). Correlation analyses show that serum TNF-alpha values significantly correlate with reperfusion values (r = 0.43, *p* < 0.01).

### 3.3. Effects of High Dose EV Treatment on Muscle Proteins following Ischemia and Reperfusion

Proteomic analysis of the muscle lysates shows that 77 proteins are differentially expressed between vehicle and high-dose EV mice (Table S2). These include 24 upregulated proteins with EV treatment and 53 downregulated with EV treatment (Figure S1). Functional enrichment analysis shows that upregulated proteins in EV-treated mice are most associated with the acute inflammatory response and wound healing (Table 3; Figure S2). Inflammatory response proteins include fibrinogen, fibronectin, and haptoglobin, whereas wound healing proteins include plasminogen, complement C3, and the most highly upregulated protein annexin a1, which is known to have anti-inflammatory effects (Table 3 and Table S2).

Functional enrichment of the downregulated proteins shows that these proteins are generally associated with myopathies (Table 4; Figure S3). For example, obscurin, plectin, synemin, and dystrophin are all decreased in muscle with EV treatment (Table 4 and Table S2).

## 4. Discussion

This study evaluated the potential application of lyophilized EVs secreted from adipose-derived stem cells to prevent ischemia-reperfusion injury. We have previously shown that a combination of trehalose and PVP can support the bioactivity of EVs during storage and that these EVs can enhance muscle cell survival after ischemia [10]. Our proteomic characterization of these EVs showed that their protein cargo is similar to cargo previously described for EVs [15,16], including EVs from fresh adipose-derived stem cells [17]. The cargo includes actin-binding proteins such as moesin and gelsolin, as well as heat-shock 90 (Hsp90) proteins. The latter are particularly relevant from the perspective of I/R injury as Hsp90 has been demonstrated to increase nitric oxide production by increasing eNOS expression [18,19]. It is certainly possible that Hsp90, among other factors not studied here, such as microRNAs, may contribute to the increased angiogenesis and perfusion others have noted with EV treatment in ischemic models [7,8,9]. Consistent with these other studies, we found enhanced perfusion with high-dose EV treatment, suggesting that a primary mechanism by which stem cell-derived EVs may impact ischemic tissue is via vasodilation. Our proteomic data indicate that annexin a1 is the most upregulated protein in muscle from mice receiving high-dose EVs. In addition to its well-established anti-inflammatory properties, annexin a1 may also increase eNOS in the microvasculature [20]. Together these results suggest that adipose-derived EVs may be useful not only for increasing perfusion in ischemic conditions but also in microvascular disorders.

The increased perfusion we observed with high-dose EVs was, however, not associated with improved muscle structure. Histological results indicate significant damage to muscle tissue compared to vehicle-treated mice. Proteomic data from the muscle of mice receiving high-dose EVs revealed decreased levels of dystrophin, plectin, obscurin, and synemin. Loss of dystrophin has previously been described after ischemia in cardiac muscle [21,22]; however, to our knowledge, ours is the first study to identify decreases in obscurin, plectin, and synemin after ischemia. Obscurin and plectin are both very large proteins involved in intermediate filament stabilization, and mutations in these proteins are known to be associated with various myopathies [23,24]. Synemin is an intermediate filament protein, and synemin mutations are associated with cardiac and skeletal muscle dysfunction [25]. The disruption of these proteins after high-dose EV treatment, along with the histological observations of muscle damage, suggest that the high-dose treatment has a significant negative effect on sarcomere integrity and muscle structure.

Previous work suggests that much of the damage associated with I/R injury is due to either prolonged ischemia or relatively uncontrolled reperfusion [26]. Thus, interventions to reduce I/R would focus on shortening the ischemic interval and/or controlling reperfusion using various approaches, such as ischemic preconditioning or gentle reperfusion [27]. Our data indicating that high-dose EVs increase reperfusion, increase tissue damage, and increase local and circulating inflammatory cytokines suggest that these outcomes may be indicative of relatively uncontrolled, excessive reperfusion. In this context, EV therapy may be more appropriate for more chronic, low-level microvasculature dysfunction rather than in the context of acute ischemia in the surgical or trauma setting. To that end, therapies that address the specific changes in mitochondria with I/R injury may be a more effective approach. These approaches might include small molecules such as mPTP inhibitors. Indeed, we [4] and others [5] have previously shown that the mPTP inhibitor NIM-811 reduces local and systemic inflammatory cytokines after I/R and reduces ischemic damage to muscle cells in vitro [4].

There are several considerations raised by our study for future investigation. The first is that lyophilization and storage, while attractive as an “off the shelf” therapeutic approach for EVs, may lead to some EV degradation. This is likely to be one reason why the lower EV dose was not as effective as the higher dose at increasing reperfusion in our study; however, a more important point to consider is that if the goal of the intervention is to improve perfusion, then lyophilized EVs may indeed be highly effective. The concern is, as noted above, that the increased level of perfusion may be relatively “uncontrolled”. A second area for future study is that we focused on the protein cargo of the EVs, but it is well established that EVs are also enriched in miRNAs. It is unknown to what extent the lyophilization and storage processes impact the miRNA cargo. It is certainly possible that alternative strategies for preservation and storage may better preserve the miRNAs and, thus, the bioactivity of the EVs. Finally, we directed our study toward the changes in skeletal muscle with EV treatment following ischemia and reperfusion, but it is likely that the EVs may impact other organs and tissues. For example, EVs injected via the tail vein are known to go to the liver, and it is not clear how they might impact liver-derived cytokines. The fact that circulating TNF-alpha increased with high-dose EV treatment, but muscle-derived TNF-alpha did not suggest that other organs, such as the liver, may be impacted by the EV treatment. A direct comparison of tail vein EV injection versus intramuscular, subcutaneous, or intraperitoneal injection would be informative with regard to this question. Future studies are expected to address some of these limitations to better characterize the potential of stem cell-derived EVs for treating ischemia-reperfusion injury.

## Figures and Tables

**Table 1:** Proteins detected in lyophilized EVs previously detected in exosomes (ExoCarta) and in fresh EVs from ADSCs [15,16].

Gene Symbol	UniProt Id	Sequence Name
Fn1 *	P11276	Fibronectin-1
Col12a1	Q60847	Collagen-1 alpha XII
Col1a2	Q01149	Collagen 1A2
Tnc	Q80YX1	Tenascin C
Lgals3bp	Q07797	Galectin 3 binding protein
Postn	Q62009	Periostin
Thbs2 *	Q03350	Thrombospondin-2
Myh9 *	Q8VDD5	Myosin-9
Lum	P51885	Lumican
Lrp1	Q91ZX7	Prolow-density lipoprotein receptor-related protein 1
Bgn	P28653	Biglycan
Gsn *	P13020	Gelsolin
Msn *	P26041	Moesin
Hsp90ab1	P11499	Heat shock protein HSP 90-beta
Vcan	Q62059	Versican
Hsp90b1	P11499	Endoplasmin
Hsp90aa1 *	P07901	Heat shock protein HSP 90-alpha
Eno1 *	P17182	Alpha-enolase
Pros1	Q08761	Vitamin K-dependent protein S (Pros1)
App	P12023	Amyloid-beta A4 protein
Lbp	Q61805	Lipopolysaccharide-binding protein
Calr	P14211	Calreticulin
Cltc *	Q68FD5	Clathrin heavy chain 1
Nid1	P10493	Nidogen 1
Fbln2	P37889	Fibulin 2
Col1a1	P11087	Collagen alpha-1 (I)

* Proteins commonly detected in exosomes from multiple cell types [15].

**Table 2:** ANOVA results from parameters examined in mice treated with vehicle or low-dose (50 ug) extracellular vesicles.

Variable	Treatment	Sex	Treatment*Sex
Reperfusion (flux units)	*p* = 0.43	0.89	0.224
Muscle TNF alpha	*p* = 0.19	<0.001	0.09
Muscle IL1 alpha	*p* = 0.09	0.68	0.39
Muscle IL6	*p* = 0.17	0.01	0.63
Serum TNF alpha	*p* = 0.87	0.35	0.16
Serum IL1 alpha	*p* = 0.58	0.72	0.22
Serum IL6	*p* = 0.40	0.23	0.72

**Table 3:** Proteins identified from functional enrichment (ToppFun) analysis of upregulated proteins (Table S2) in the EV-treated hindlimb related to acute inflammatory response and wound healing. Fold-change (FC) and *p*-values are shown for each gene.

Acute Inflammatory Response		Wound Healing	
Gene symbol	Gene name	Gene symbol	Gene name
Fga (FC = 2.74 ***)	fibrinogen alpha chain	Fga (FC = 2.74 ***)	fibrinogen alpha chain
Apcs (FC = 2.54 **)	amyloid P component, serum	Fgb (FC = 2.9 ***)	fibrinogen beta chain
Orm1 (FC = 3.3 ***)	orosomucoid 1	Apcs (FC = 2.54 **)	amyloid P component, serum
Serpinc1 (FC = 2.48 **)	serpin family C member 1	S100a9 (FC = 2.9 **)	S100 calcium-binding protein A9
C3 (FC = 2.37 **)	complement C3	Serpinc1 (FC = 2.48 **)	serpin family C member 1
Fn1 (FC = 5.0 **)	fibronectin 1	C3 (FC = 2.37 **)	complement C3
Serpinf2 (FC = 2.6 ***)	serpin family F member 2	Fgg (FC = 4.1 ***)	fibrinogen gamma chain
Pzp (FC = 4.6 **)	PZP alpha-2-macroglobulin like	Plg (FC = 3.5 **)	plasminogen
Hp (FC = 3.4 **)	haptoglobin	Fn1 Fn1 (FC = 5.0 **)	fibronectin 1
Itih4 (FC = 2.8 **)	inter-alpha-trypsin inhibitor heavy chain 4	Serpinf2 (FC = 2.6 ***)	serpin family F member 2
		Anxa1 (FC = 6.7 ***)	annexin A1
		Gsn (FC = 4.7 **)	gelsolin

** *p* < 0.01, *** *p* < 0.001.

**Table 4:** Proteins identified from functional enrichment (ToppFun) analysis of downregulated muscle structural proteins (Table S2) in the EV-treated hindlimb. Fold-change (FC) and *p*-values are shown for each gene.

Muscle Structural Proteins	
Gene symbol	Gene name
Tnni2 (FC = 0.69 **)	troponin I, fast skeletal muscle
Dmd (FC = 0.48 **)	dystrophin
Plec (FC = 0.44 ***)	plectin
Obscn (FC = 0.42 **)	obscurin
Synm (FC = 0.43 ***)	synemin

** *p* < 0.01, *** *p* < *0*.001.

## Data Availability

Data are provided as Supplementary Materials.

## Supplementary Materials

The following supporting information can be downloaded at: https://www.mdpi.com/article/10.3390/cells12040557/s1, Figure S1: 77 significant proteins; Figure S2: Significant Terms For: GO: Biological Process; Figure S3: Significant Terms For: Disease; Table S1: LyophEVMassSpec. Table S2: CONTvTRT.
