# Supplementary figures and images for: Lyophilized Extracellular Vesicles from Adipose-Derived Stem Cells Increase Muscle Reperfusion but Degrade Muscle Structural Proteins in a Mouse Model of Hindlimb Ischemia-Reperfusion Injury
Source: Cells. 2023 Feb 9;12(4):557. doi: 10.3390/cells12040557 (PMC9953864; doi:10.3390/cells12040557)

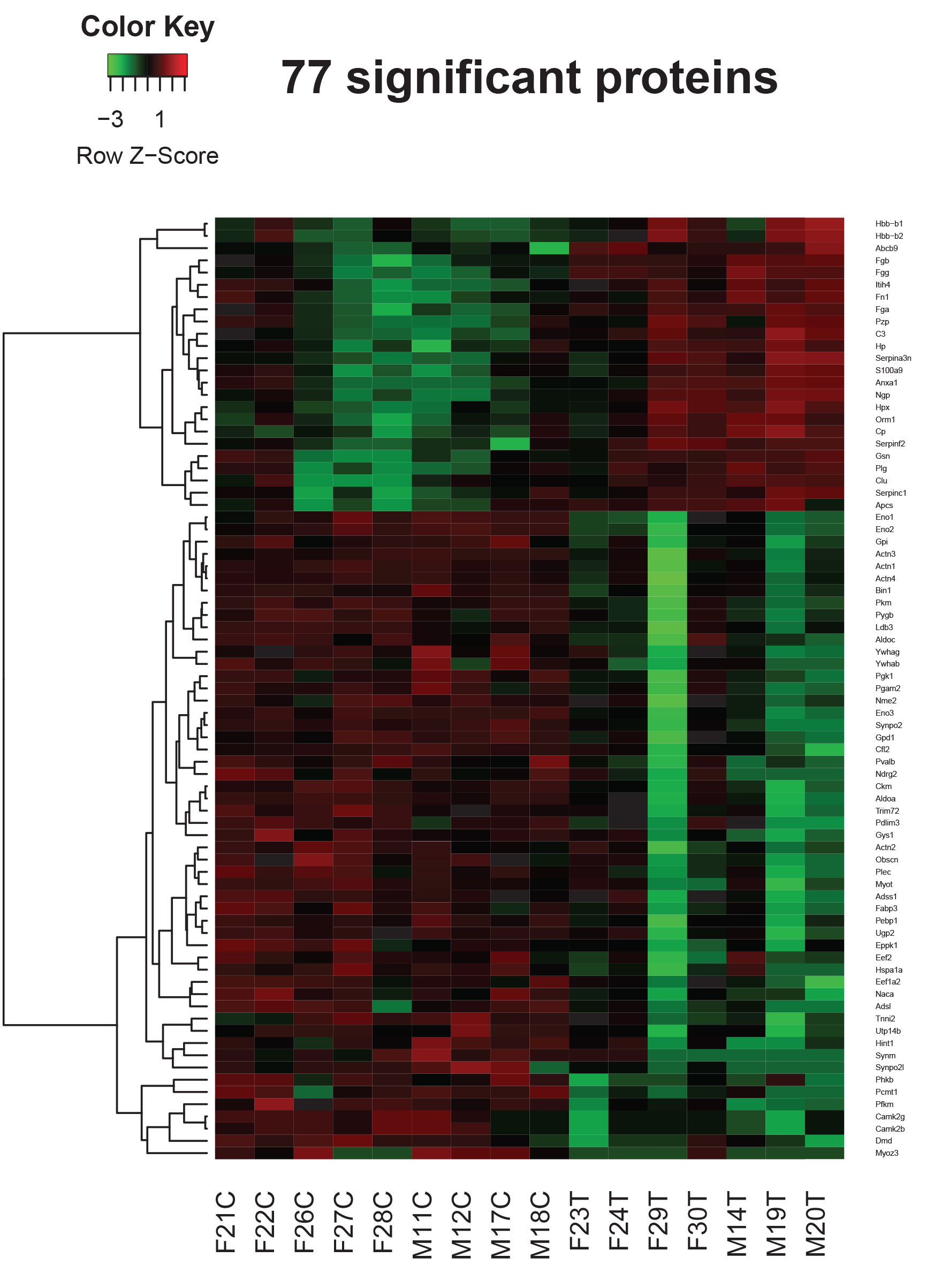

Supplement: Supplementary file 1 [file cells-12-00557-s001.zip › Supp Figure S1-HeatmapProteomics.tif]

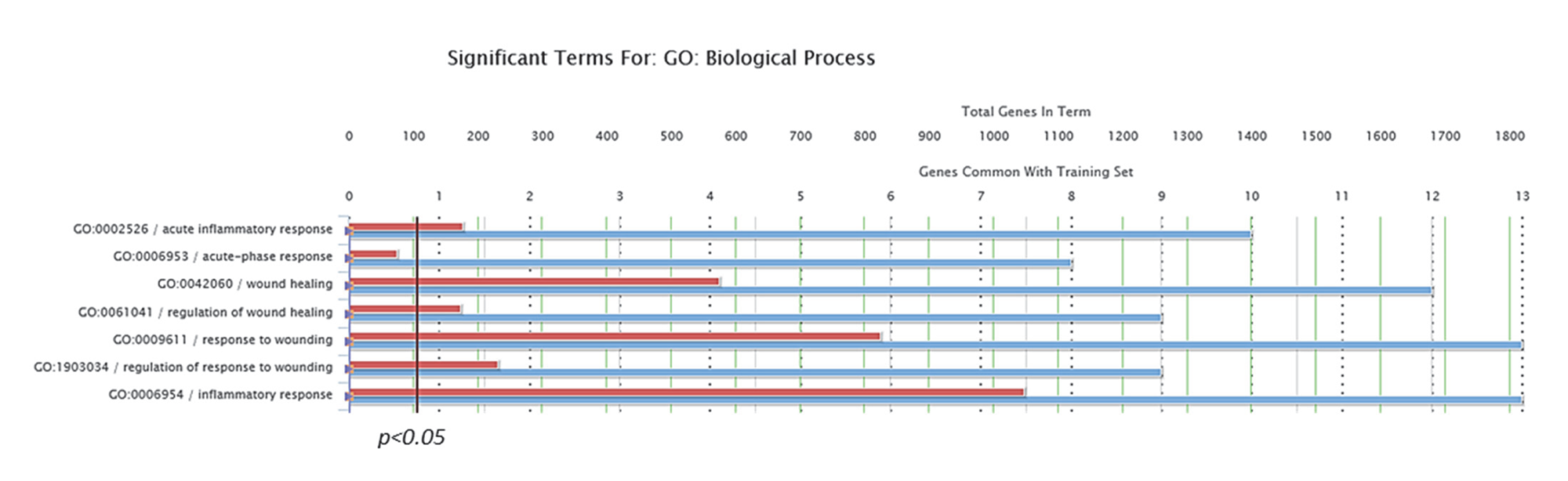

Supplement: Supplementary file 1 [file cells-12-00557-s001.zip › Supp Figure S2-FunctEnrichUp.tif]

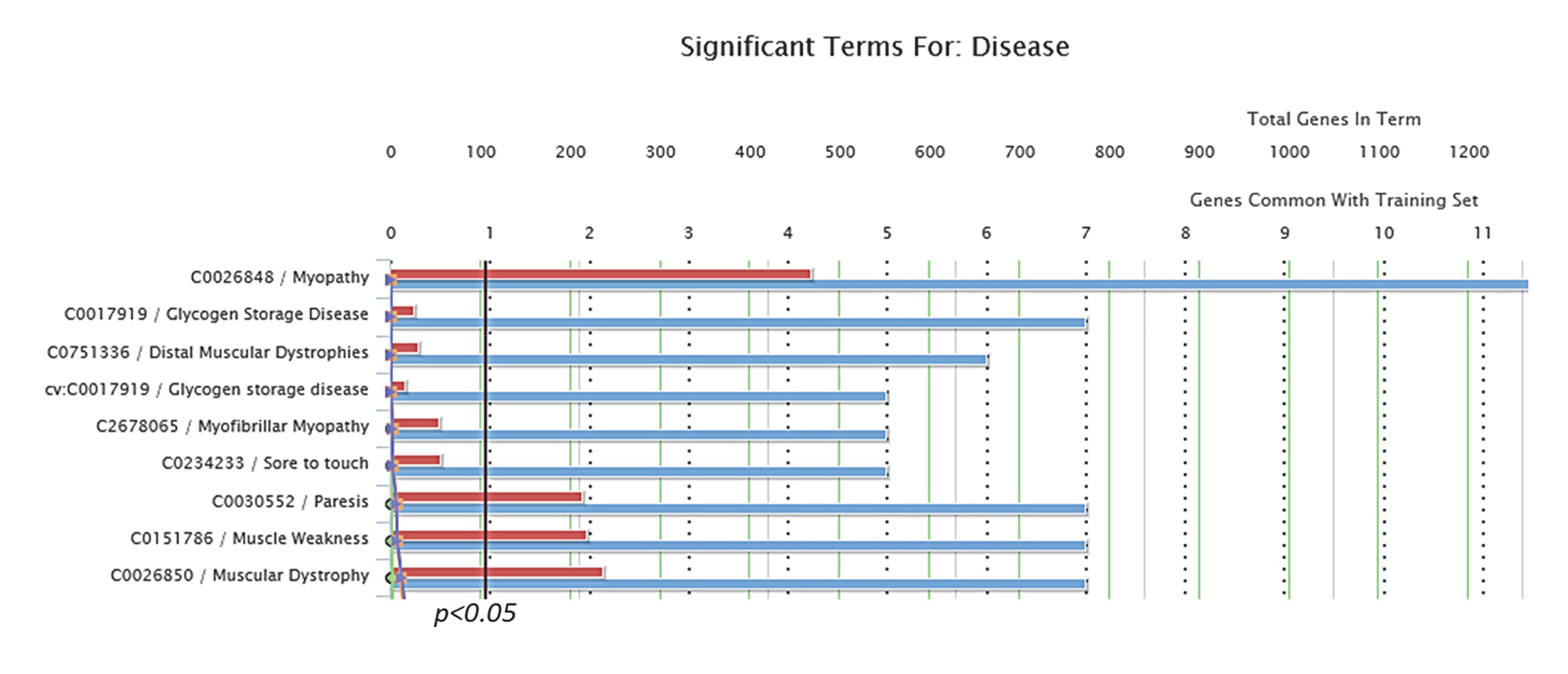

Supplement: Supplementary file 1 [file cells-12-00557-s001.zip › Supp Figure S3-FunctEnrichDown.tif]
